# Poly[(μ_3_-camphorato-κ^3^ *O*:*O*′:*O*′′)(2-methyl-1*H*-imidazole-κ*N* ^3^)zinc(II)]

**DOI:** 10.1107/S1600536810007105

**Published:** 2010-03-03

**Authors:** Haochen Shi, Chunjie Li, Feng Gao, Jingyan Chen, Fei Han

**Affiliations:** a Department of Physics Education, Changchun Normal University, 667 Changji Highway (North), Erdao District, Jilin Province 130032, People’s Republic of China

## Abstract

In the title compound, [Zn(C_10_H_14_O_4_)(C_4_H_6_N_2_)]_*n*_, each Zn^II^ ion is coordinated by one N atom from one 2-methyl-1*H*-imidazole ligand and three O atoms from two camphorate (cap) ligands in a distorted tetra­hedral geometry. In one of the cap ligands, one methyl group is disordered between positions 1 and 3 in a 0.518 (12):0.482 (12) ratio. Each cap ligand bridges three Zn^II^ ions, forming two-dimensional layers, which inter­act further *via* N—H⋯O hydrogen bonds.

## Related literature

For general background to coordination polymers based on camphoric acid, see: Zhang *et al.* (2007 ▶).
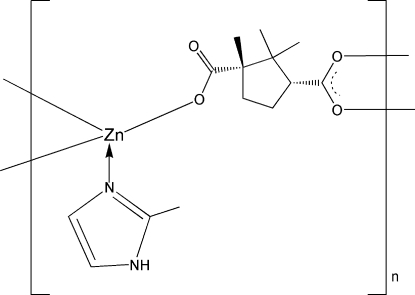


## Experimental

### 

#### Crystal data

[Zn(C_10_H_14_O_4_)(C_4_H_6_N_2_)]
*M* *_r_* = 345.69
Monoclinic, 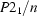

*a* = 12.098 (2) Å
*b* = 10.438 (5) Å
*c* = 12.873 (2) Å
β = 111.700 (5)°
*V* = 1510.4 (8) Å^3^
*Z* = 4
Mo *K*α radiation
μ = 1.64 mm^−1^
*T* = 293 K
0.31 × 0.25 × 0.21 mm

#### Data collection

Bruker APEX CCD area-detector diffractometer
Absorption correction: multi-scan (*SADABS*; Sheldrick, 1996 ▶) *T*_min_ = 0.57, *T*_max_ = 0.72
9848 measured reflections
3004 independent reflections
2433 reflections with *I* > 2σ(*I*)
*R*_int_ = 0.041

#### Refinement

*R*[*F* ^2^ > 2σ(*F* ^2^)] = 0.049
*wR*(*F* ^2^) = 0.121
*S* = 1.08
3004 reflections
200 parameters
H-atom parameters constrained
Δρ_max_ = 1.13 e Å^−3^
Δρ_min_ = −0.41 e Å^−3^

### 

Data collection: *SMART* (Bruker, 1998 ▶); cell refinement: *SAINT* (Bruker, 1998 ▶); data reduction: *SAINT*; program(s) used to solve structure: *SHELXS97* (Sheldrick, 2008 ▶); program(s) used to refine structure: *SHELXL97* (Sheldrick, 2008 ▶); molecular graphics: *SHELXTL-Plus* (Sheldrick, 2008 ▶); software used to prepare material for publication: *SHELXL97*.

## Supplementary Material

Crystal structure: contains datablocks global, I. DOI: 10.1107/S1600536810007105/cv2697sup1.cif

Structure factors: contains datablocks I. DOI: 10.1107/S1600536810007105/cv2697Isup2.hkl

Additional supplementary materials:  crystallographic information; 3D view; checkCIF report

## Figures and Tables

**Table 1:** Hydrogen-bond geometry (Å, °)

*D*—H⋯*A*	*D*—H	H⋯*A*	*D*⋯*A*	*D*—H⋯*A*
N2—H2⋯O3^i^	0.86	1.86	2.722 (5)	176

Symmetry code: (i) 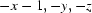.

**Fig. 1.**
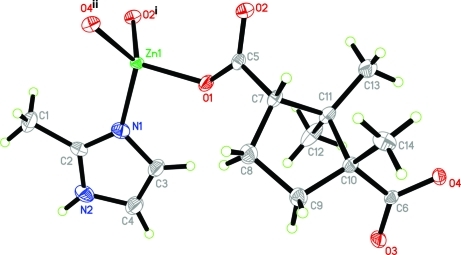
A portion of the crystal structure of (I), showing the atomic numbering and 30% probability displacement ellipsoids [symmetry codes: (i) -x, -y, -z; (ii) x+1/2, -1/2-y, z+1/2]. Only major parts of disordered atoms are shown.

## supplementary crystallographic information

### Comment

Coordination polymers based on camphoric acid (cap) have received intense interests because of their potential applications as functional solid materials, as well as their fascinating framework structures (Zhang *et al.*, 2007). We report here the synthesis and structure of the title compound (I).

In (I) (Fig. 1), each Zn^II^ atom is four-coordinated by one nitrogen atom from one 2-methyl-1*H*-imidazole (mid) ligand and three oxygen atoms from two different camphorate anions (cap) in a distorted tetrahedral geometry. Each cap ligand bridges three Zn^II^ atoms to form a two-dimensional layer structure. Further, the N—H···O H-bonding interactions (Table 1) stabilize the structure of (I).

### Experimental

A mixture of ZnCl^.^2H_2_O (1 mmol), NaOH (1 mmol), D-camphoric acid (1 mmol) and 2-methyl-1*H*-imidazole (1 mmol) in water (12 ml) was heated to 140 °C for three days in a 25 ml Teflon-lined stainless steel vessel under autogenous pressure. Subsequently, it was cooled to room temperature. Then, single crystals of (I) were obtained.

### Refinement

All H atoms were positioned geometrically (C—H = 0.93-0.98 Å; N—H = 0.86 Å) and refined as riding, with U_iso_(H)=1.2-1.5U_eq_ of the carrier atom. In cap ligand, one methyl group was treated as disordered between positions 1 (C14) and 3 (C14') in a ratio 0.518 (12):0.482 (12), respectively.

### Crystal data

**Table:**

[Zn(C_10_H_14_O_4_)(C_4_H_6_N_2_)]	*F*(000) = 720
*M**_r_* = 345.69	*D*_x_ = 1.520 Mg m^−^^3^
Monoclinic, *P*2_1_/*n*	Mo *K*α radiation, λ = 0.71069 Å
Hall symbol: -P 2yn	Cell parameters from 3004 reflections
*a* = 12.098 (2) Å	θ = 2.0–26.2°
*b* = 10.438 (5) Å	µ = 1.64 mm^−^^1^
*c* = 12.873 (2) Å	*T* = 293 K
β = 111.700 (5)°	Block, colourless
*V* = 1510.4 (8) Å^3^	0.31 × 0.25 × 0.21 mm
*Z* = 4	

### Data collection

**Table:**

Bruker APEX CCD area-detector diffractometer	3004 independent reflections
Radiation source: fine-focus sealed tube	2433 reflections with *I* > 2σ(*I*)
graphite	*R*_int_ = 0.041
φ and ω scans	θ_max_ = 26.2°, θ_min_ = 2.0°
Absorption correction: multi-scan (*SADABS*; Sheldrick, 1996)	*h* = −14→14
*T*_min_ = 0.57, *T*_max_ = 0.72	*k* = −12→9
9848 measured reflections	*l* = −15→15

### Refinement

**Table:**

Refinement on *F*^2^	Primary atom site location: structure-invariant direct methods
Least-squares matrix: full	Secondary atom site location: difference Fourier map
*R*[*F*^2^ > 2σ(*F*^2^)] = 0.049	Hydrogen site location: inferred from neighbouring sites
*wR*(*F*^2^) = 0.121	H-atom parameters constrained
*S* = 1.08	*w* = 1/[σ^2^(*F*_o_^2^) + (0.0392*P*)^2^ + 5.0738*P*] where *P* = (*F*_o_^2^ + 2*F*_c_^2^)/3
3004 reflections	(Δ/σ)_max_ < 0.001
200 parameters	Δρ_max_ = 1.13 e Å^−^^3^
0 restraints	Δρ_min_ = −0.41 e Å^−^^3^

### Special details

**Table:**

Geometry. All esds (except the esd in the dihedral angle between two l.s. planes) are estimated using the full covariance matrix. The cell esds are taken into account individually in the estimation of esds in distances, angles and torsion angles; correlations between esds in cell parameters are only used when they are defined by crystal symmetry. An approximate (isotropic) treatment of cell esds is used for estimating esds involving l.s. planes.
Refinement. Refinement of *F*^2^ against ALL reflections. The weighted *R*-factor wR and goodness of fit *S* are based on *F*^2^, conventional *R*-factors *R* are based on *F*, with *F* set to zero for negative *F*^2^. The threshold expression of *F*^2^ > σ(*F*^2^) is used only for calculating *R*-factors(gt) etc. and is not relevant to the choice of reflections for refinement. *R*-factors based on *F*^2^ are statistically about twice as large as those based on *F*, and *R*- factors based on ALL data will be even larger.

### Fractional atomic coordinates and isotropic or equivalent isotropic displacement parameters (Å^2^)

**Table:**

	*x*	*y*	*z*	*U*_iso_*/*U*_eq_	Occ. (<1)
C1	−0.1477 (5)	0.3115 (6)	0.2334 (4)	0.0469 (14)	
H1A	−0.1842	0.3736	0.2655	0.070*	
H1B	−0.1009	0.3547	0.1980	0.070*	
H1C	−0.0974	0.2562	0.2911	0.070*	
C2	−0.2432 (4)	0.2327 (4)	0.1474 (4)	0.0295 (10)	
C3	−0.3337 (4)	0.0952 (5)	0.0210 (5)	0.0423 (13)	
H3	−0.3476	0.0292	−0.0308	0.051*	
C4	−0.4157 (5)	0.1593 (6)	0.0419 (5)	0.0483 (14)	
H4	−0.4975	0.1475	0.0082	0.058*	
C5	−0.0941 (4)	−0.1910 (4)	−0.0050 (3)	0.0238 (9)	
C6	−0.4416 (4)	−0.4768 (4)	−0.2027 (3)	0.0216 (9)	
C7	−0.1627 (4)	−0.3138 (4)	−0.0087 (3)	0.0248 (9)	
H7	−0.1055	−0.3841	0.0182	0.030*	0.518 (12)
C14'	−0.0756 (10)	−0.4218 (9)	0.0453 (10)	0.042 (3)	0.482 (12)
H14A	−0.0215	−0.4318	0.0070	0.063*	0.482 (12)
H14B	−0.1187	−0.5002	0.0406	0.063*	0.482 (12)
H14C	−0.0317	−0.4015	0.1224	0.063*	0.482 (12)
C8	−0.2402 (5)	−0.3118 (5)	0.0598 (4)	0.0426 (13)	
H8A	−0.2719	−0.2265	0.0603	0.051*	
H8B	−0.1948	−0.3371	0.1363	0.051*	
C9	−0.3432 (4)	−0.4089 (5)	0.0035 (4)	0.0325 (11)	
H9A	−0.3379	−0.4809	0.0527	0.039*	
H9B	−0.4199	−0.3678	−0.0140	0.039*	
C10	−0.3275 (4)	−0.4530 (4)	−0.1027 (4)	0.0241 (9)	
H10	−0.2816	−0.5328	−0.0860	0.029*	0.482 (12)
C14	−0.2562 (8)	−0.5802 (9)	−0.0776 (8)	0.034 (3)	0.518 (12)
H14D	−0.1835	−0.5686	−0.0144	0.051*	0.518 (12)
H14E	−0.2383	−0.6046	−0.1416	0.051*	0.518 (12)
H14F	−0.3027	−0.6461	−0.0614	0.051*	0.518 (12)
C11	−0.2500 (4)	−0.3472 (5)	−0.1293 (4)	0.0289 (10)	
C12	−0.3273 (5)	−0.2323 (5)	−0.1839 (4)	0.0477 (15)	
H12A	−0.3682	−0.2029	−0.1371	0.072*	
H12B	−0.3844	−0.2569	−0.2555	0.072*	
H12C	−0.2781	−0.1647	−0.1935	0.072*	
C13	−0.1875 (5)	−0.3910 (6)	−0.2067 (5)	0.0504 (15)	
H13A	−0.1376	−0.4632	−0.1741	0.076*	
H13B	−0.1397	−0.3223	−0.2169	0.076*	
H13C	−0.2458	−0.4151	−0.2778	0.076*	
N1	−0.2211 (4)	0.1414 (4)	0.0891 (3)	0.0356 (9)	
N2	−0.3575 (4)	0.2492 (4)	0.1247 (4)	0.0424 (11)	
H2	−0.3898	0.3040	0.1545	0.051*	
O1	−0.1397 (3)	−0.0873 (3)	0.0066 (3)	0.0305 (7)	
O2	0.0054 (3)	−0.2004 (3)	−0.0152 (3)	0.0268 (7)	
O3	−0.5387 (3)	−0.4312 (3)	−0.2099 (3)	0.0360 (8)	
O4	−0.4297 (3)	−0.5450 (3)	−0.2795 (2)	0.0276 (7)	
Zn1	−0.06957 (4)	0.06945 (4)	0.08772 (4)	0.02053 (16)	

### Atomic displacement parameters (Å^2^)

**Table:**

	*U*^11^	*U*^22^	*U*^33^	*U*^12^	*U*^13^	*U*^23^
C1	0.050 (3)	0.050 (3)	0.034 (3)	−0.018 (3)	0.007 (2)	−0.005 (2)
C2	0.031 (3)	0.028 (2)	0.034 (3)	0.006 (2)	0.018 (2)	0.006 (2)
C3	0.027 (3)	0.044 (3)	0.049 (3)	−0.002 (2)	0.006 (2)	−0.014 (3)
C4	0.034 (3)	0.049 (3)	0.058 (4)	−0.003 (3)	0.012 (3)	−0.015 (3)
C5	0.025 (2)	0.025 (2)	0.017 (2)	−0.0031 (18)	0.0029 (17)	0.0006 (17)
C6	0.022 (2)	0.017 (2)	0.026 (2)	−0.0033 (17)	0.0086 (18)	0.0000 (17)
C7	0.024 (2)	0.024 (2)	0.021 (2)	−0.0043 (18)	0.0019 (18)	0.0012 (17)
C14'	0.044 (6)	0.016 (5)	0.066 (8)	−0.002 (5)	0.021 (6)	0.008 (5)
C8	0.049 (3)	0.048 (3)	0.036 (3)	−0.016 (3)	0.022 (3)	−0.010 (2)
C9	0.033 (3)	0.035 (3)	0.028 (2)	0.000 (2)	0.010 (2)	−0.001 (2)
C10	0.024 (2)	0.021 (2)	0.026 (2)	−0.0017 (17)	0.0079 (18)	0.0007 (18)
C14	0.029 (5)	0.038 (6)	0.034 (5)	0.016 (4)	0.010 (4)	0.013 (4)
C11	0.028 (2)	0.031 (3)	0.025 (2)	−0.0091 (19)	0.0054 (19)	0.0008 (19)
C12	0.044 (3)	0.038 (3)	0.040 (3)	−0.016 (3)	−0.009 (2)	0.016 (2)
C13	0.047 (3)	0.065 (4)	0.047 (3)	−0.023 (3)	0.027 (3)	−0.020 (3)
N1	0.033 (2)	0.035 (2)	0.042 (2)	0.0060 (18)	0.0166 (19)	0.0021 (19)
N2	0.039 (2)	0.045 (3)	0.049 (3)	0.014 (2)	0.022 (2)	0.000 (2)
O1	0.0250 (16)	0.0240 (17)	0.0390 (18)	−0.0056 (13)	0.0076 (14)	−0.0079 (14)
O2	0.0243 (16)	0.0238 (17)	0.0314 (17)	−0.0002 (13)	0.0091 (13)	0.0064 (13)
O3	0.0220 (16)	0.042 (2)	0.0415 (19)	0.0034 (15)	0.0094 (14)	−0.0164 (16)
O4	0.0251 (16)	0.0278 (17)	0.0286 (16)	0.0008 (13)	0.0083 (13)	−0.0088 (13)
Zn1	0.0181 (2)	0.0174 (3)	0.0269 (3)	0.0014 (2)	0.00920 (19)	−0.0012 (2)

### Geometric parameters (Å, °)

**Table:**

C1—C2	1.513 (7)	C8—H8B	0.9700
C1—H1A	0.9600	C9—C10	1.519 (6)
C1—H1B	0.9600	C9—H9A	0.9700
C1—H1C	0.9600	C9—H9B	0.9700
C2—N1	1.300 (6)	C10—C14	1.550 (9)
C2—N2	1.315 (6)	C10—C11	1.567 (6)
C3—C4	1.304 (7)	C10—H10	0.9800
C3—N1	1.405 (6)	C14—H14D	0.9600
C3—H3	0.9300	C14—H14E	0.9600
C4—N2	1.399 (7)	C14—H14F	0.9600
C4—H4	0.9300	C11—C12	1.523 (7)
C5—O1	1.249 (5)	C11—C13	1.527 (7)
C5—O2	1.262 (5)	C12—H12A	0.9600
C5—C7	1.519 (6)	C12—H12B	0.9600
C6—O3	1.238 (5)	C12—H12C	0.9600
C6—O4	1.269 (5)	C13—H13A	0.9600
C6—C10	1.521 (6)	C13—H13B	0.9600
C7—C8	1.506 (6)	C13—H13C	0.9600
C7—C14'	1.524 (11)	N1—Zn1	1.988 (4)
C7—C11	1.560 (6)	N2—H2	0.8600
C7—H7	0.9800	O1—Zn1	1.957 (3)
C14'—H14A	0.9600	O2—Zn1^i^	1.968 (3)
C14'—H14B	0.9600	O4—Zn1^ii^	1.926 (3)
C14'—H14C	0.9600	Zn1—O4^iii^	1.926 (3)
C8—C9	1.561 (7)	Zn1—O2^i^	1.968 (3)
C8—H8A	0.9700		
			
C2—C1—H1A	109.5	C9—C10—C6	115.8 (4)
C2—C1—H1B	109.5	C9—C10—C14	108.5 (5)
H1A—C1—H1B	109.5	C6—C10—C14	107.3 (5)
C2—C1—H1C	109.5	C9—C10—C11	105.5 (4)
H1A—C1—H1C	109.5	C6—C10—C11	111.1 (3)
H1B—C1—H1C	109.5	C14—C10—C11	108.5 (5)
N1—C2—N2	113.0 (5)	C9—C10—H10	108.1
N1—C2—C1	123.8 (4)	C6—C10—H10	108.1
N2—C2—C1	123.2 (4)	C14—C10—H10	0.8
C4—C3—N1	109.4 (5)	C11—C10—H10	108.1
C4—C3—H3	125.3	C10—C14—H14D	109.5
N1—C3—H3	125.3	C10—C14—H14E	109.5
C3—C4—N2	107.1 (5)	H14D—C14—H14E	109.5
C3—C4—H4	126.5	C10—C14—H14F	109.5
N2—C4—H4	126.5	H14D—C14—H14F	109.5
O1—C5—O2	124.1 (4)	H14E—C14—H14F	109.5
O1—C5—C7	118.3 (4)	C12—C11—C13	107.6 (4)
O2—C5—C7	117.6 (4)	C12—C11—C7	110.9 (4)
O3—C6—O4	122.3 (4)	C13—C11—C7	113.6 (4)
O3—C6—C10	122.7 (4)	C12—C11—C10	109.9 (4)
O4—C6—C10	115.0 (4)	C13—C11—C10	114.4 (4)
C8—C7—C5	114.9 (4)	C7—C11—C10	100.3 (3)
C8—C7—C14'	102.6 (5)	C11—C12—H12A	109.5
C5—C7—C14'	109.4 (5)	C11—C12—H12B	109.5
C8—C7—C11	104.3 (4)	H12A—C12—H12B	109.5
C5—C7—C11	112.4 (3)	C11—C12—H12C	109.5
C14'—C7—C11	112.8 (6)	H12A—C12—H12C	109.5
C8—C7—H7	108.3	H12B—C12—H12C	109.5
C5—C7—H7	108.3	C11—C13—H13A	109.5
C14'—C7—H7	6.3	C11—C13—H13B	109.5
C11—C7—H7	108.3	H13A—C13—H13B	109.5
C7—C14'—H14A	109.5	C11—C13—H13C	109.5
C7—C14'—H14B	109.5	H13A—C13—H13C	109.5
H14A—C14'—H14B	109.5	H13B—C13—H13C	109.5
C7—C14'—H14C	109.5	C2—N1—C3	104.7 (4)
H14A—C14'—H14C	109.5	C2—N1—Zn1	132.0 (4)
H14B—C14'—H14C	109.5	C3—N1—Zn1	123.4 (3)
C7—C8—C9	106.4 (4)	C2—N2—C4	105.9 (4)
C7—C8—H8A	110.5	C2—N2—H2	127.0
C9—C8—H8A	110.5	C4—N2—H2	127.0
C7—C8—H8B	110.5	C5—O1—Zn1	131.7 (3)
C9—C8—H8B	110.5	C5—O2—Zn1^i^	123.6 (3)
H8A—C8—H8B	108.6	C6—O4—Zn1^ii^	116.8 (3)
C10—C9—C8	105.7 (4)	O4^iii^—Zn1—O1	115.33 (13)
C10—C9—H9A	110.6	O4^iii^—Zn1—O2^i^	98.14 (12)
C8—C9—H9A	110.6	O1—Zn1—O2^i^	119.76 (13)
C10—C9—H9B	110.6	O4^iii^—Zn1—N1	123.56 (15)
C8—C9—H9B	110.6	O1—Zn1—N1	95.81 (15)
H9A—C9—H9B	108.7	O2^i^—Zn1—N1	105.57 (15)

Symmetry codes: (i) −*x*, −*y*, −*z*; (ii) *x*−1/2, −*y*−1/2, *z*−1/2; (iii) *x*+1/2, −*y*−1/2, *z*+1/2.

### Hydrogen-bond geometry (Å, °)

**Table:**

*D*—H···*A*	*D*—H	H···*A*	*D*···*A*	*D*—H···*A*
N2—H2···O3^iv^	0.86	1.86	2.722 (5)	176

Symmetry codes: (iv) −*x*−1, −*y*, −*z*.
